# Antecedents for blockchain technology-enabled sustainable agriculture supply chain

**DOI:** 10.1007/s10479-021-04423-3

**Published:** 2021-12-06

**Authors:** Kirti Nayal, Rakesh D. Raut, Balkrishna E. Narkhede, Pragati Priyadarshinee, Gajanan B. Panchal, Vidyadhar V. Gedam

**Affiliations:** 1 Department of Operations and Supply Chain Management, National Institute of Industrial Engineering (NITIE), Vihar Lake, Powai, Mumbai, Maharashtra 400087 India; 2 Chaitanya Bharathi Institute of Technology (CBIT), Gandipet, Hyderabad, Telangana 500075 India; 3 Operations & Information Management, Aston Business School, Birmingham, United Kingdom; 4 Environmental Engineering and Management, National Institute of Industrial Engineering (NITIE), #610, Level 6, ALB Building, Powai, Mumbai, 400087 India

**Keywords:** Blockchain technology (BLCT), Sustainable supply chain performance (SSCP), Agricultural-food supply chain (ASC), Structural equation modeling (SEM)

## Abstract

Blockchain can solve the problems that the agriculture supply chain (ASC) is facing to achieve sustainable growth. In a nation like India, blockchain application in the supply chain is still new; therefore, supply chain players need a better understanding and awareness of blockchain through valuable insights. This article aims to study the mediating role of blockchain technology adoption (BLCT) for sustainable supply chain performance (SSCP). This study investigates the influence of numerous factors such as green and lean practices, supply chain integration, supply chain risk, performance expectancy, top management support, cost, internal and external environmental conditions, regulatory support, and innovation capability on BLCT adoption. A sample of 316 respondents from Indian ASC industries was collected, and structural equation modeling (SEM) was used. This study's outcomes show that green and lean practices, supply chain integration, supply chain risks, internal and external conditions, regulatory support, innovation capability, and cost positively influence BLCT adoption. Moreover, BLCT positively influences sustainable agriculture supply chain performance. This article is valuable for policymakers, managers, service providers, researchers, and academicians to understand the role of factors in influencing BLCT and BLCT's role in improving sustainable supply chain performance (SSCP).

## Introduction

The agriculture supply chain includes the suppliers, processors, distributors, and consumers, in which the final product is either consumed by humans or animals, and the raw material is produced in farms (Miranda-Ackerman & Azzaro-Pantel, 2017). In recent years, ASC has been receiving significant attention for sustainable growth consisting of best agricultural practices, well being of all stakeholders, and protection of the environment (Castro & Swart, 2017; Dentoni & Peterson, 2011). ASC is also under severe pressure from numerous consumer organizations, agriculture firms, social and environmental activists, and policymakers to achieve sustainable performance (Allaoui et al., 2018). ASC has many issues such as a low level of industrialization, ineffective supply chain management (SCM), lack of managerial skills, and inefficient information sharing, resulting in a low level of supply chain (SC) visibility (Luthra et al., 2018). The significant challenges that need to be addressed to achieve sustainable performance in ASC are lack of small farmers' integration, lack of strict food quality and safety regulations, and information quality (Naik & Suresh, 2018). A BLCT based data management system will reduce the chances of food frauds and adulteration, thus increasing sustainable performance. BLCT can act as a digital platform providing authentic information on the provenance of agricultural products (Ge et al., 2017). The agricultural export from India is likely to reach the target of 60 billion US dollars by 2022. In India, agriculture is the primary source of income for about 58% of the population. The export of agricultural and affiliated products reached 41.25 billion US dollars in 2020–21 (“Agriculture in India,” 2021). The Indian ASC faces problems of meeting the ever-growing population’s demand, poor storage infrastructure, poor quality leading to more food losses, and a high number of intermediaries leading to delayed transactions (Ritchie et al., 2018; Balaji & Arshinder, 2016).

BLCT supports sustainability by utilizing its four capabilities: 1. Reduction in food recall due to its better traceability nature, 2. Determination of accurate Carbon emission and tax because of its transparent and traceable nature, 3. Facilitate recycling by encouraging people to participate in deposit-based recycling programs, 4. Increase the efficiency of emission trading schemes by decreasing fraud and improving the system (Saberi et al., 2018). These capabilities also increase consumers' and SC players' awareness of business sustainability practices while improving SC performance (Kouhizadeh & Sarkis, 2018). There exist many challenges to blockchain implementation in the supply chain to improve traceability, visibility, and transparency hampering BLCT implementation. These challenges are not only limited to the high cost of investment, scalability, interoperability, bidirectionality, data privacy and security, scaling latency, time verification, confusions created by competing technologies, and lack of regulations legislations (Swan, 2015). Apart from these technological issues, there are also organizational, cultural, and behavioral hurdles to completely exploit the BLCT’s potential. For example, lack of management and government support, lack of organizational policies and culture of blockchain adoption, lack of employees' skills and knowledge, and digital literacy (Khaqqi et al., 2018; Mendling et al., 2018). The decentralization characteristic of BLCT that means data cannot be stored at one point in the chain is a big challenge to make SC sustainable. Eventually, BLCT use in SC makes it high-performance-oriented, more energy-efficient, cost-effective, transparent, efficient, and effective in utilizing resources. Thus, BLCT will help establish sustainable SC (Yadav & Singh, 2020a; Kshetri, 2018). In literature, researchers have discussed the role of various factors on BLCT adoption (e.g., Kouhizadeh et al., 2021; Nandi et al., 2020; Wamba et al., 2020) and on SSCP (e.g., Han & Huo, 2020; Asadi et al., 2020; Orji & Liu, 2020), separately. Many authors have highlighted the potential of BLCT to improve SC's sustainability because of its unique capabilities and characteristics (Esmaeilian et al., 2020; Saberi et al., 2019; Kouhizadeh & Sarkis, 2018; Iansiti & Lakhani, 2017). Thus, the objective of this research article is to examine the effect of identified factors on BLCT adoption and then further study the effect of BLCT adoption on SSCP.

As an emerging technology, BLCT is still in its developmental stage due to the challenges in its adoption, and practitioners and researchers are trying BLCT implementation in the supply chain area (Queiroz & Wamba, 2019; Kamble et al., 2019). The authors in extant literature have explored the effect of BLCT on SC performance, BLCT critical success factors for sustainable SC, perceived usefulness of BCLT, performance measurement model for transparency of BCLT based system, BLCT enabled critical sustainability factors, conceptual model for BLCT adoption for green and sustainable SC, user perception of BLCT adoption, critical success factor for BLCT adoption, challenges and opportunities of BLCT adoption. However, the literature lacks exhaustive research on essential success factors on BLCT adoption and sustainable supply chain performance. Therefore, this study proposes the following research questions to address the gap:

- RQ1 What are the factors that affect BLCT adoption in ASC?

- RQ2 Does BLCT adoption affect sustainable supply chain performance?

- RQ3 Does BLCT play a mediating role in between the factors affecting its adoption and SSCP?

With sustainable performance (SP) consideration, the BLCT adoption model can help policymakers and practitioners understand BLCT better with empirical evidence. Therefore, this study aims to study BLCT's mediating effect on SSCP of the agri-food sector in India by considering the factors affecting blockchain adoption based on technological, organizational, and environmental (TOE) and unified theory of acceptance and use of technology (UTAUT) theory with slight variations. The factors and subfactors of the proposed model were identified from the literature survey, and SEM was used for analyzing these factors.

This study's outline is organised as follows: Section 2 presents the study's existing review study and background. Section 3 discussed the conceptual framework and hypothesis development for the study. Section 4 describes the research methodology. Section 5 and 6 represent the empirical findings and the discussion and implications of the study. The last section of the study discussed the conclusion and limitations, and future scope.

## Literature review

In the available literature focused on BLCT adoption and its role in improving supply chain performance (SCP) and sustainability. The literature was searched from ‘Scopus’ and ‘Web of Science’ (WoS). ‘Sustainability’, ‘performance’, ‘blockchain’, ‘supply chain’, and ‘SEM’ were used as keywords for searching articles on an online database. No time limit was set, and searches were limited to reviews and articles. By combining all keyword combinations, removing repeated articles, conference, and non-peer-reviewed articles, and after thorough reading and detailed analysis of papers, 16 articles were finalized for this study, excluding multi-criteria decision making (MCDM) techniques, case study, and conceptual papers. The relevant literature found is discussed in the next sections based on which research gap is written as follows:

### BLCT and SEM

Li and Fang (2021) explored the factors influencing information resource sharing intention through the view of unanimity of BLCT’s perception, and Kamble et al. (2019) developed a model for user perception on the adoption of BLCT by using SEM in the supply chain. Karamchandani et al. (2020) examined whether the perceived usefulness of permissioned BLCT comes from the knowledge of BLCT benefits or publicity. The SEM was used to test the proposed hypothesis among the perceived benefits, perceived usefulness, and incremental profitability.

Some authors have studied the impact of BLCT on SCP, firm performance, financial performance, or sustainable performance by using partial least squares (PLS-SEM) or SEM and found a significant positive impact (e.g., Paul et al., 2021; Masudin et al., 2021; Khan et al., 2021a, 2021b; Wamba et al., 2020; Kim & Shin, 2019; Sheel & Nath, 2019).

Benzidia et al. (2021) explored the role of BLCT in collaborative supplier management to increase the innovation capabilities of buying firms by using PLS-SEM. Wamba and Queiroz (2020) explored the determinants of BLCT diffusion in supply chains by using PLS-SEM. Kim and Shin (2019) investigated the impact of BLCT on SC partnership growth and efficiency and thus on SCP by using SEM. Wamba et al. (2020) examined the potential impact of BLCT on SCP by using SEM. Where SCP is crucially affected by BLCT enabled transparency. Sheel and Nath (2019) showed that BLCT could improve SCP by improving trust, agility, alignment, adaptability, and competitiveness. Queiroz et al. (2021) and Queiroz and Wamba (2019) developed a model for the adoption behaviour of BLCT in the supply chain by utilizing PLS-SEM. Wong et al. (2020b) also discovered the behavioural intention to adopt BLCT by examining the effect of critical factors on its adoption and validating the proposed model through reliability and validity. Also, Wong et al. (2020a) studied the effect of critical factors on BLCT adoption using partial least squares-artificial neural network (PLS-ANN) analysis.

### SEM and ASC

A few authors have used SEM for their study in ASC. Paul et al. (2021) and Masudin et al. (2021) performed their study for BLCT by utilizing SEM and PLS-SEM, respectively. However, Nayal et al. (2021) performed for artificial intelligence (AI) by using SEM. Paul et al. (2021) investigated the impact of BLCT on the sustainable performance of organic tea SC. The findings showed that BLCT adoption has a significant and positive impact on sustainable performance by improving SC's transparency and reliability. Masudin et al. (2021) determined the effect of managerial initiatives on adopting traceability systems of cold food chain and the effect of traceability systems on food cold chain performance during the COVID-19 and found a significant positive relationship for both. Nayal et al. (2021) studied the factors impacting AI adoption and investigated AI's influence on SC risk mitigation. Nayal et al. (2021) discussed that the variables discussed are technological factors, organizational factors, process factors, environmental factors, information sharing, SC integration, AI, and SC risk mitigation. This study showed that process factors, information sharing, and SC integration influences AI adoption, and AI further influences SC risk mitigation. The remaining variables have a non-significant negative relation with AI adoption.

### Antecedents of blockchain adoption

It is clear from the literature that factors such as SC integration (Nandi et al., 2020; Yadav et al., 2020), SC risk (Kamble et al., 2020; Wong et al., 2020b), internal and external environment conditions (Kouhizadeh et al., 2021; Wong et al., 2020b; Queiroz et al., 2021), regulatory support (Wong et al., 2020a, 2020b), performance expectancy (Stranieri et al., 2021; Wamba et al., 2020; Wong et al., 2020b; Queiroz et al., 2021; Queiroz & Wamba, 2019), top management support (Kouhizadeh et al., 2021; Wong et al., 2020a), innovation capability (Nandi et al., 2020) and cost (Yadav & Singh, 2020a; Nandi et al., 2020; Kamble et al., 2020; Wong et al., 2020a; Yadav & Singh, 2020b) may affect BLCT adoption in the supply chain. The effect of green and lean practices on BLCT adoption for sustainable performance is neglected by researchers; although, green and lean practices play an essential role in improving sustainable performance (Raut et al., 2019). Blockchain improves collaboration by providing real-time information sharing, improving the system's transparency, trust, and security (Stranieri et al., 2021; Iansiti & Lakhani, 2017). BLCT assists in mitigating risks by effective management of supply and demand, SC resources, and inventory (Ivanov et al., 2019). The internal and external environmental conditions such as availability of resources including technical skills and expertise, presence of advanced information-sharing technology system, intention to adopt blockchain technology, competitive pressure, the influence of customs, culture, and people can influence BLCT adoption (Kouhizadeh et al., 2021; Shi & Yan, 2016). Regulatory support (RESU) provides legal certainty to the users of BLCT by implementing guidelines related to data protection and its use for transparent SC processes. This will also improve the trust of SC players for BLCT use (Wong et al., 2020b). The particular and robust policies and laws for BLCT adoption result in quick adoption (Shi & Yan, 2016). Performance expectancy (PERE) is also a suitable catalyst for technology adoption (Batara et al., 2017). It influences behavioural intention to adopt BLCT (Francisco & Swanson, 2018). Some top managers fail to provide support for the adoption of disruptive technology like blockchain. BLCT has gained practitioners' attention, but managers still have insufficient knowledge about blockchain, making managers hesitant to adopt it (Kouhizadeh et al., 2021). Innovation capability, including adopting new technology like BLCT for transforming and reconfiguring current resources, can mitigate SC risks by reducing truckload and thus reducing cost, leading to improvement in supply chain performance (SCP) (Wang et al., 2020; Teece et al., 1997). The blockchain removes the mediator, human error, paperwork with the help of a shared database, secured system, and improved decision making (Yadav & Singh, 2020b). This leads to a reduction in overall cost (Nandi et al., 2020). However, BLCT implementation incurs huge implementation costs (Kamble et al., 2020).

### Antecedents of SSCP

Authors in the extant literature have shown the relation of supply chain integration (SUCI) (Han & Huo, 2020; Shee et al., 2018), RESU (Asadi et al., 2020; Orji & Liu, 2020), PERE (Yadav & Singh, 2020b), top management support (TMSU) (Orji & Liu, 2020; Shee et al., 2018), innovation capability (INNC) (Asadi et al., 2020; Rathore et al., 2020) and cost (Yadav & Singh, 2020b) with SSCP. Integrative supply chain management may help firms achieve sustainable SC performance by promoting firms' internal motivation to implement green SC integration practices and better manage resources to achieve green goals. Suppliers can share going green costs and increase economic performance, whereas consumers can push demands related to social issues by providing authentic product feedback (Han & Huo, 2020). Higher the SUCI means higher the SCP in quality, cost delivery, and flexibility (Banchuen et al., 2017), enhancing SSCP. Environmental regulations support green innovation related to the execution of environment-friendly packaging, reuse and recycling of materials, eco-labeling, and less production of toxics and waste, which can positively influence sustainable SC firm performance (Asadi et al., 2020). Government and regulatory legislations also act as drivers for promoting sustainability goals (Orji & Liu, 2020). Top management initiatives, sufficient and efficient support, participation, and willingness decide the success of sustainable strategies related to green and lean innovations and initiatives to promote sustainability (Orji & Liu, 2020; Shee et al., 2018). Innovation capabilities can improve SSCP by supporting green and lean practices in the supply chain (Asadi et al., 2020; Rathore et al., 2020). In literature, supply chain risk (SUCR), green and lean practices, and internal and external environment conditions (IEEC) are not empirically explored concerning sustainable supply chain performance. Internal and external environment influences green culture and practices by supporting top management leadership in implementing green strategies (Li et al., 2019), thus influencing SSCP. Green innovation, integration, and practices affect sustainable performance (Asadi et al., 2020; Muduli et al., 2020; Han & Huo, 2020; Miemczyk & Luzzini, 2019). Lean practices positively influence sustainable performance by reducing waste and increasing delivery performance (Orji & Liu, 2020; Rathore et al., 2020). Supply chain risk related to climate change uncertainty, disasters, and uncertainty of demand can lead to a decrement in the sustainable performance of the supply chain.

### Research gap

Empirical studies based on SEM are performed in agriculture, manufacturing, service, multisector, warehousing, and logistics supply chains. More researchers have carried out their studies on multisector (e.g., Khan et al., 2021a, 2021b; Li & Fang, 2021; Wamba & Queiroz, 2020; Queiroz et al., 2021; Kim & Shin, 2019), and few have carried out in agriculture sector (Paul et al., 2021; Masudin et al., 2021). Authors have used PLS-SEM, SEM, and PLS-ANN methodology in their research in available literature related to empirical studies of BLCT adoption in SC. In literature, some studies have focussed on variables related to the conceptual model of this study (Benzidia et al., 2021; Wong et al., 2020a, 2020b; Wamba & Queiroz, 2020; Queiroz et al., 2021; Queiroz & Wamba, 2019). The studies relevant to variables of our conceptual models are carried out in manufacturing, service industry, or not in any sector-specific supply chain and by utilizing PLS-ANN or PLS-SEM. Benzidia et al. (2021) studied internal integration, BLCT, buyer’s innovation variables and found a positive relation between BLCT and internal integration. The authors have also found that BLCT significantly mediates the relation between internal capabilities and buyer’s innovation. Wong et al. (2020b) discussed performance expectancy, regulatory support, facilitating conditions, and BLCT and revealed that facilitating conditions positively influence BLCT intention to adopt. Whereas regulatory support moderates the effect of facilitating conditions on BLCT adoption. Wamba and Queiroz (2020) studied the relation between top management support and BLCT adoption and found it positively significant for India but not for the United States (US). Queiroz et al. (2021) studied BLCT adoption, performance expectancy, and facilitating conditions and concluded that facilitating conditions are critically decisive in predicting BLCT adoption but performance expectancy is not decisive. Queiroz and Wamba (2019) studied facilitating conditions, performance expectancy, and BLCT adoption. The findings of this study revealed that performance expectancy positively influences the behavioural intention to adopt blockchain for both Indian and US cases. At the same time, facilitating conditions determine both the behavioural intention and expectation for blockchain in the case of the US but not in the Indian case. Wong et al. (2020a) studied cost, regulatory support, top management support, and BLCT. The findings revealed that cost has a significant negative affect on behavioural intention. Whereas top management support and regulatory support are insignificant related to behavioural intention to adopt BLCT.

Paul et al. (2021) and Masudin et al. (2021) focussed on sustainable performance and SCP in ASC by using SEM and PLS-SEM. Therefore, in ASC, enough empirical literature is lacking on BLCT and its adoption factors. A few have discussed the BLCT effect on performance (e.g., Paul et al., 2021; Masudin et al., 2021). However, not on factors affecting intention to adopt BLCT. However, some literature is available on adoption factors for BLCT in other sectors’ supply chains. The available literature has not focussed on factors of BLCT adoption derived from combining both UTAUT and TOE theory in one model. Although, Nayal et al. (2021) studied model for AI adoption by combining TOE and OIPT theory. This study first identifies the factors that influence BLCT adoption and then examines the role of BLCT in improving the SSCP of ASC. This research article also provides a conceptual model for measuring the SSCP of BLCT-enabled ASC.

## Conceptual framework and hypothesis development

**Fig. 1:**
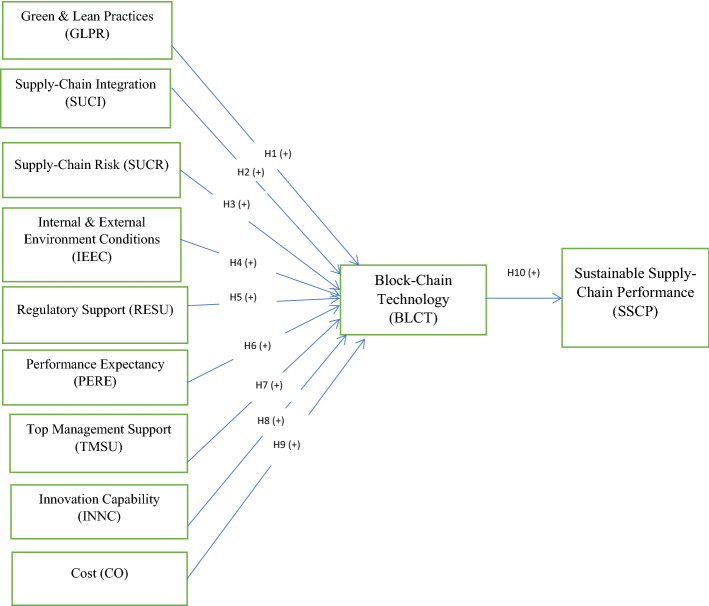
Conceptual framework of the proposed blockchain application model

This article anticipated a research model based on the UTAUT (Venkatesh et al., 2003, 2012) and TOE (Tornatzky et al., 1990) with slightly modified factors for blockchain technology adoption. This model has added cost, green and lean practices, and supply chain risk variables (Wang et al., 2020; Chiarini et al., 2020; Wong et al., 2020a; Raut et al., 2019). Additionally, the present need for machining ASC is sustainable to make the model more suitable for blockchain technology adoption for risk management in an uncertain scenario. The model consists of nine factors, considering that they may significantly influence blockchain technology. The measurable variable sustainable supply chain risk performance is included for measuring the sustainable performance (SP) of blockchain-enabled ASC. Moreover, many authors have used UTAUT (Queiroz et al., 2021; Queiroz & Wamba, 2019; Kamble et al., 2019) and TOE (Kouhizadeh et al., 2020; Bai & Sarkis, 2020; Wong et al., 2020a) constructs previously in their research model. Based on these studies, we have prepared our proposed model. The proposed conceptual framework and the detailed proposed factors and sub-factors are shown in Fig. 1, and the details are shown in the appendix (Table 6).

### Green and lean practices (GLPR)

GLPR complement each other because they are combined to achieve sustainable targets in SCM. GLPR, when combined in SCM, then it provides an ability to the firms to eliminate all types of waste, especially environmental waste. This waste elimination results in eco-friendly products produced at a low cost. GLPR improves ecological and operational performance (Inman & Green, 2018). Many authors have used and emphasized the need to consider lean and green practices for examining blockchain adoption to determine whether these practices are reasons to implement BLCT or not (Chiarini et al., 2020; Raut et al., 2019). Therefore, in this study, lean and green practices are considered variables to understand blockchain adoption for improving the SP of ASC. Efficient resource utilization, emission reduction, the energy efficiency of process and design, application of lean tools and practices, and lean and green technology come under the variable of lean and green practices.

#### H1

*Lean and Green practices positively influence blockchain technology adoption.*

### Supply chain integration (SUCI)

SUCI is described as ‘a way of working in coordination and partnership for the product, information, cash, and data flow by considering all SC players' requirement towards the same goal of achieving the higher service quality and profit’ (Ataseven & Nair, 2017). Many authors have concluded ‘integration’ as a significant factor for industry 4.0 technologies adoption (Chiarini et al., 2020; Raut et al., 2019; Singh et al., 2019). In this study, ‘integration’ is considered a feature of SC where the firm understands the requirement of SC players, shares information, coordinates, collaborates, makes strategic alliances for a common goal, exists public-private partnership, and shares innovation and knowledge among the stakeholders.

#### H2

*Supply Chain Integration positively influence blockchain technology adoption*

### Supply chain risk (SUCR)

SUCR is defined as a threat that can disrupt the daily process and activities and thus hinder the planning in supply chains (Flynn et al., 2016). This study SUCR variable comprises low consequences, negative impacts, and errors that badly affect the ASC. The purpose of using the supply chain risk variable is that it is a critical variable in uncertainty and the industry 4.0 era (Wang et al., 2020). This study has included three types of risks under the supply chain risk variable based on available extensive literature. Company side risk consists of the risks that can potentially disrupt information and product flow (Ellegaard, 2008). Delays in product delivery and pickups and inadequate storage and delivery capacity come under company-side risks. Customer side risks usually happen from the consumer-related activities and mistakes from the inquiry of products to the delivery of products and improvement in order. In our study, we have included demand volatility and poor or inaccurate forecasting under customer-side risks. The third supply chain risks category, environment side risk, occurs due to the external environment and events that are not part of the supply chain network. Environmental side risk is unavoidable and plays a crucial role in SC (Wang et al., 2020). Therefore, road and border closures and volatile fuel prices are included under the environment side risk category in this study.

#### H3

*Supply chain risk positively influences Blockchain technology adoption.*

### Internal and external environment conditions (IEEC)

IEEC refers to the SC environment's facilitating conditions, which are defined as the variable that comprises facilitating resources, environmental conditions, and organizational support to support technology use and application (Venkatesh et al., 2003, 2012). The role of internal and external environmental conditions for this study is discussed by studying the influence of the availability of resources, technical expertise, knowledge, and experience, the advanced information-sharing requirement for a better quality of information, trust in blockchain technology, competitive pressure for improving the profitability and reputation through the strategic decision making and social influence of customs, culture, and people that forces to implement BLCT through the belief of society in the importance of BLCT (Karamchandani et al., 2020; Wong et al., 2020a; Queiroz et al., 2021).

#### H4

*Internal and external environment conditions positively influence blockchain technology adoption*

### Regulatory support (RESU)

Regulatory support indicates rules and regulations significant for promoting blockchain technology implementation (Shi & Yan, 2016). Sufficient and relevant regulatory support from the regulatory authorities and government makes implementation fast (Wong et al., 2020a; Shi & Yan, 2016). Financial support from relevant regulatory bodies also plays an essential role in BLCT implementation (Wong et al., 2020a). Regulatory support is crucial for blockchain implementation studies (Wong et al., 2020a, 2020b). Regulatory uncertainties and other intellectual property issues and compliance are top priorities that need to be solved for BLCT adoption (Wong et al., 2020a). Regulatory support helps develop trust and affects infrastructure implementation for BLCT adoption, thus affecting technology readiness and supporting conditions (Wong et al., 2020b).

#### H5

*Regulatory support positively influences blockchain technology adoption.*

### Performance expectancy (PERE)

PERE is described as “the degree to which a user trusts that using the technology-enabled system will help in a job or task performance” (Venkatesh et al., 2003). This variable is also recognized as crucial in realizing technology adoption by various authors (Batara et al., 2017). In this study, performance expectancy is a set of believed items to improve ASC's performance and are used as performance measures such as productivity, risk reduction, overall quality improvement, and speed of tasks (Queiroz et al., 2021; Wong et al., 2020b).

#### H6

*Performance expectancy positively influence blockchain technology adoption*

### Top management support (TMSU)

TMSU is defined as “the degree to which the TMSU understands the significance of blockchain and participates in its adoption” (Ooi et al., 2018). Top management commitment and active participation play an essential role in successfully implementing any technology (Dubey et al., 2018). Suppose the advantages of blockchain adoptions enable it to provide profit by overcoming the cost challenges. In that case, top management also supports the employees in learning and implementing BLCT (Wong et al., 2020a). Many authors use this variable in their BLCT adoption model. Therefore, TMSU is a critical factor in BLCT adoption.

#### H7

*Top management support positively influences blockchain adoption.*

### Innovation capability (INNC)

INNC is described as the administration's ability to convert ideas and knowledge into new systems, processes, services, and products for organizational benefits (Yang, 2012). Innovation capability in the supply chain is considered a more significant capability to redevelop operational capabilities to achieve the best SC operations and mitigate SC risks (Teece et al., 1997). In our study, innovation capability can apply the SC innovation to provide innovative and technical solutions to the problem, adjust a dynamic SC environment, and improve SCP by providing standardized and straightforward operations in the supply chain.

#### H8

*Innovation capability positively influences blockchain technology adoption Integration.*

### Cost (CO)

Cost is defined as money that needs to be paid and spent on implementing BLCT technology in SC (Hanif et al., 2010). The cost of technology that needs to be paid for implementation plays a vital role in determining the intention to adopt blockchain and deciding its usefulness by the upper management (Dwivedi et al., 2016). A higher cost of technology implementation generally acts as a barrier to implementing technology and its systems (Shi & Yan, 2016). Usually, a new technology implementation incurs higher costs due to specific and more users' training to get familiar with complicated technology like blockchain (Gallardo et al., 2018; Museli & Jafari Navimipour, 2018). The cost related to blockchain adoption is not straightforward and complex to calculate. There is also a need to determine the transactions, operations, and maintenance costs in blockchain-enabled SCM (Wong et al., 2020a).

#### H9

*Cost directly influences blockchain technology in agriculture supply chain management.*

### Blockchain technology (BLCT)

Blockchain is a mediating variable in our research model that influences sustainable supply chain performance. The blockchain variable is about blockchain capabilities, features, relative advantages, and its positive impacts on the system. This mediating variable consists of a total of 13 items. Trust or reliability means sharing credible or trustworthy information among supply chains due to a decentralized database (Queiroz et al., 2021; Karamchandani et al., 2020). The compatibility of BLCT with other technologies such as the internet of things (IoT), cyber-physical system (CPS), industry 4.0 also plays a vital role in blockchain benefits (Queiroz et al., 2021). Transparency refers to the automatic and identical accessibility of real-time information to all the SC participants. Attempts to damage the authenticity of the information can be easily detected and traced (Underwood, 2016). Blockchain-enabled transparency improves the visibility of SC, the efficiency of collaboration, and reduces risks. It also improves the reliability of the whole SC information of items and cash flows (Kim & Shin, 2019; Kouhizadeh & Sarkis, 2018). Traceability refers to the ability when blockchain application facilitates the SC to trace back and track the data from the procurement to the delivery (Jeppsson & Olsson, 2017). Immutability indicates that the BLCT ability enabled SC information to be unchangeable over time (Tran et al., 2017). It comes with the nature of a decentralized database in blockchain, which reduces the vulnerability to cyber hacks and frauds of data in blockchain-enabled networks (Kshetri, 2018). Stakeholders of SC conveniently sign a smart contract to make a consensus related to SC transactions and document exchanges. Any transaction and transfer occur digitally in the smart contract (Zheng et al., 2020; Kamble et al., 2019).

Complexity refers to the difficulty of using and implementing technology (Bhattacharya & Wamba, 2015). To overcome the complexity of BLCT technology, there is a need to integrate it into existing systems. Blockchain adoption can be hindered by its low transaction speed and immature security (Saberi et al., 2018). Disintermediation: the integrity of data cannot be secured by intermediaries but by SC's blockchain itself. This capability of blockchain is known as disintermediation (Michelman, 2017). Blockchain automatically generates verifiable records for all transaction-related information, increasing accountability (Hofmann et al., 2018). The records generated in the BLCT system related to all transaction information can be checked for accuracy, authenticity, and correctness (Chang et al., 2019). The acentric database facility makes the BLCT-enabled SC fault-free and helps create a trust trail known as Auditability (Wijaya et al., 2017). The blockchain improves SC visibility and accountability by guaranteeing data integration (Wang et al., 2019a). The facilities of unique ring signatures and cryptographic private keys ensure data security and user privacy (Nakamoto, 2008). Blockchain utilizes an open database through which metadata is distributed to different nodes or computers that are data cannot be collected at one point for communication; this feature is known as an acentric database (Kamble et al., 2019). Assigning digital tokens or fingerprints to each asset or product in a BLCT enabled SC to ensure the asset's last-mile connectivity throughout the SC. This results in data accountability by securing data sources or provenance (Wang et al., 2019a).

#### H10

*Blockchain technology positively influences the sustainable supply chain performance of the agri-food sector.*

### Sustainable supply chain performance (SSCP)

SSCP is a measuring variable in our study based on the triple bottom line (TBL) sustainability approach, including environmental, economic, and social indicators for measuring sustainable performance. Economic indicators include SC overall cost (i.e., production cost, transaction cost, transportation and distribution cost, capacity change cost), environmental costs such as energy cost, and profitability or sales revenue. Ecological indicators include reducing environmental impact related to reducing negative impact or externalities through emissions, e-waste, inefficient resource utilization, etc. Reducing food waste and losses using efficient green technologies and supply chain practices also comes under environmental indicators (El Bilali & Allahyari, 2018; Allaoui et al., 2018). Our study includes the number of jobs created, the empowerment of farmers and small-scale producers, and workforce stability under the social indicator of sustainability (El Bilali & Allahyari, 2018; Allaoui et al., 2018; Tsang et al., 2018). Additionally, the food safety and security item have been added under the social indicator of SSCP.

## Research methodology

### Data collection method and demography profile

A questionnaire was developed meticulously with seven industry people and five professors for the proposed model. Seventy-one items were proposed with the help of previous literature and expert opinion in supply chain and operations. The developed questionnaire items were finalized after performing a pilot study with 110 sample respondents. The items for all variables were measured on a 7-point Likert scale. The instrument was also pre-tested through a pilot study with the academicians and industry experts and was repetitively modified to ensure the reliability and validity of the content. Lastly, the total items were reduced to 67 in the proposed model after the pilot study.

7-point Likert scale was utilized for the questionnaire survey design of the proposed conceptual model. The questionnaire was responded to by the consultants, managers, and engineers in India's agri-food industries dealing with innovative technologies implementation projects. “Innovative technologies implementation projects” refer to the projects intended to upgrade and update current technological systems and practices to increase business efficiency and productivity. Random and convenient sampling methods were used for primary data collection. Centre for Monitoring Indian Economic (CMIE) and the Indian Institution of Industrial Engineering (IIIE) database were used to derive respondents from 160 industries of the ASC sector from all over India. The data collection process was started in May 2020 and ended in August 2020. Due to lockdown and maintaining the social distance as per the Indian government guidelines in the COVID-19 era, respondents were approached through e-mail and telephonic calls. To increase the survey response rate, respondents were also approached personally wherever required based on convenience. The responses were collected from the professional who has at least two and a half years of experience in the industry and handles the innovative projects of emerging technology implementation. A total of 550 respondents were approached, out of which 373 responded, and 316 were valid responses. Three hundred sixteen valid responses are from 115 agro-industries, including fresh fruits and vegetables, the beverage industry, and the dairy industry. Table 1 presents the demographic profile of respondents. Respondents with working experience of 10-15 were the most (29.43%). Interestingly female respondents (55.69%) were more than male. The undergraduate respondents were the highest in numbers (40.50%).

**Table 1:** Demographic profile

Items		N (316)	%age
Type of agro-industry	FFVs	134	42.40
	Beverage	112	35.44
	Dairy	70	22.15
	Total	316	100
Age	25–35	132	41.77
	36–55	109	34.49
	56–75	75	23.73
	Total	316	100
Gender	Male	140	44.30
	Female	176	55.69
	Total	316	100
Educational qualification	UG	128	40.50
	PG	118	37.34
	Ph.D.	70	22.15
	Total	316	100
Years of experience	0–5	65	20.56
	5–10	87	27.53
	10–15	93	29.43
	15–20	71	22.46
	Total	316	100
Designation	Executives	105	33.23
	Managers	78	24.68
	Senior managers	51	16.14
	Technology service providers	52	16.45
	Technical consultants	30	9.49
	Total	316	100

### Measurement method

A three-step statistical method is used for hypotheses testing, including ‘exploratory factor analysis (EFA)’, ‘confirmatory factor analysis (CFA)’, and SEM. EFA, a statistical method, helps in determining the interrelationships between the model variables. It helps generate a more transparent construct model by examining the nature or pattern of constructs and reducing the number of constructs from a large set of latent constructs (Williams et al., 2010; Hair et al., 1995). To eliminate the EFA method's limitations, CFA is used to refine and validate the constructs (Ahire et al., 1996). The main advantage of CFA application in SEM for more clarity is that the validity of the expected construct model can be analysed on more than one goodness-of-fit indices (Chan et al., 2007).

SEM is applied by analyzing moment structures (AMOS)-20.0 software (Gerbing & Anderson, 1988). To evaluate the CFA model's notable change, there is a need to calculate loading estimates and path coefficients. The same value of loading estimates means the proposed construct model is valid and has no problem. This study's SEM model is based on Kline's rules (2015), MacCallum and Browne (1993). The AMOS software is used as it is equipped with all drawing tools to create and examine SEM path diagrams (Chan et al., 2007) and is capable of analysing SPSS files (Mangla et al., 2020). Path diagrams are a significant base for SEM because they present the relationships between the variables (Ullman and Bentler, 2012). In our study, SEM with factor analysis is used to analyze BLCT application constructs' effect on the sustainable supply chain performance of agri-food industries. SEM evaluates unexplored and explored constructs of BLCT application. EFA, CFA, and SEM integrated approaches justify a linear relationship as the first data analysis set.

SEM is an umbrella concept, and a structural mediation model is a part of it to check the causal relationships. Mediation is often used to provide a more accurate explanation for the antecedent's causal effect on the dependent variable. The mediator is usually the variable that is the missing link in a chain of causation (Lowry & Gaskin, 2014). The mediation effect is based upon the following three conditions:

- Full Mediation (Only Indirect Effect)
- Partial Mediation (Both Direct and Indirect Effect)
- No Mediation (No Indirect Effect)

## Empirical findings

### EFA

The qualitative and quantitative investigation method was applied to explore the SSCP of agri-food industries with the mediating effect of BLCT technology. 11 constructs with 68 items in the model were evaluated using a two-stage approach. Constructs were defined with the respective item dimension in the first stage. The validity and reliability of new indicator variables were assessed in the first stage (Bohrnstedt et al., 1983; Cronbach, 1951). A questionnaire based on the 7-point Likert scale was formed to study the proposed research model in the second stage. Bartlett’s test and Kaiser-Meyer-Olkin (KMO) were performed to check the data suitability for construct structure. KMO values nearer to 1 indicate that data is suitable for factor analysis (FA). Barlett’s Test significance (*p*) should be < 0.05 for a 95% confidence level (Hair et al., 1995). ‘KMO’ and ‘*p*’ values were found as 0.851 and 0.00 successively, which were acceptable. Principal Component Analysis (PCA) was used as an extraction method and Varimax with Kaiser Normalization as a rotation method.

The rotated component matrix linked up after six iterations. High loading (> 0.5) was obtained for all constructs. The greatest loading was obtained of “SUCI” (0.965), and the lowest loading was obtained of “IEEC” (0.634). No cross-loading was found for any variable. Therefore, CFA can be performed based on underlying relations between variables to confirm the relational structure.

### CFA

**Fig. 2:**
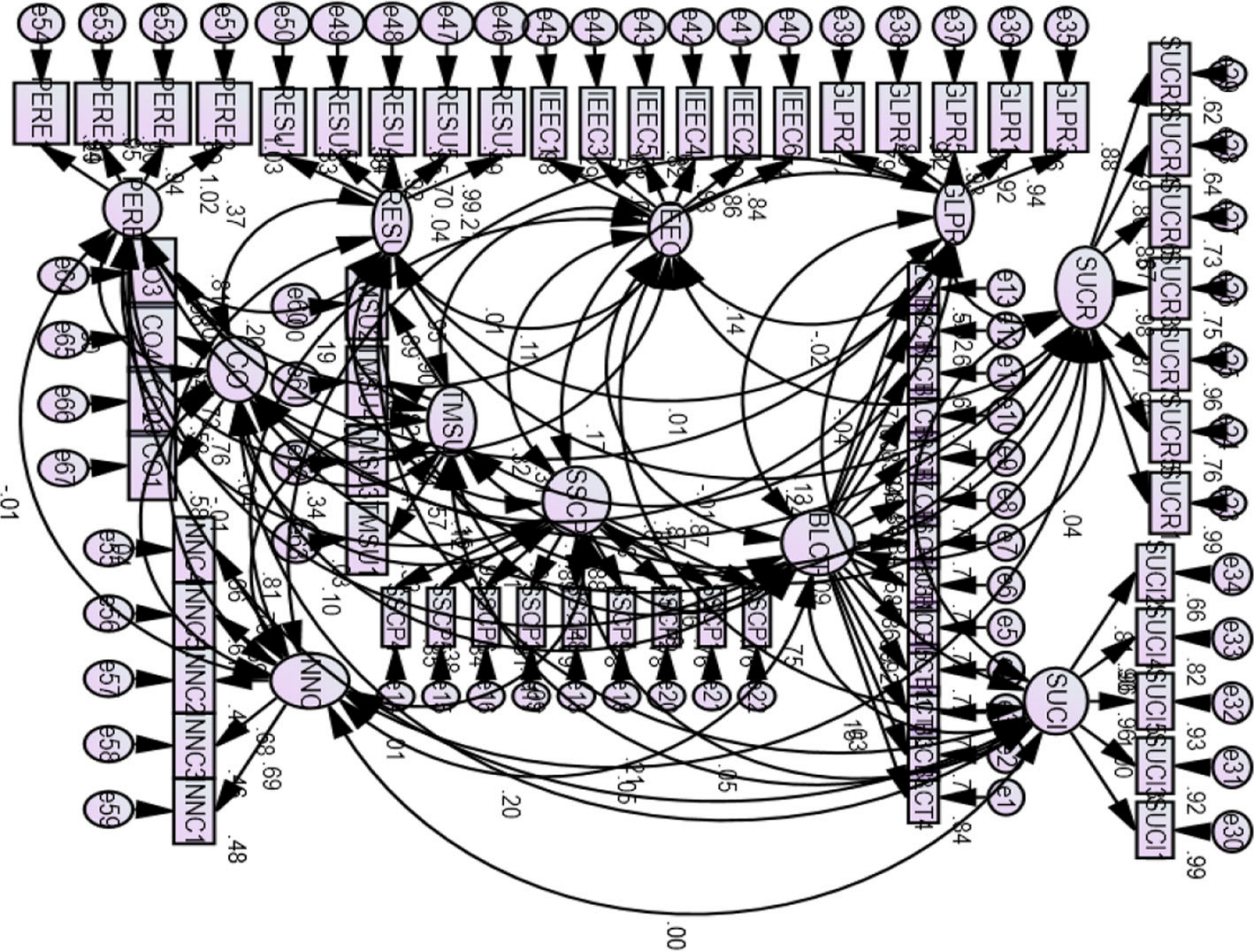
CFA path diagram

CFA was performed with nine variables for BLCT technology and one variable for SSCP of agri-food industries. All constructs were allowed to fit explicitly. The sustainable supply chain performance of ASC has nine items. The construct “blockchain technology” has more than ten items. The findings of CFA show that RMSEA (0.065) was greater than 0.05, the Chi-Square test value was 2.329 (< 3.0), and NFI (0.809) was greater than 0.8. CFI (0.881) and GFI (0.726) were lower than 0.95, within the allowable limit. Thus, we conclude that the Goodness of Fit statistics exhibits allowable findings for the data sample. The CFA figure is shown in the appendix (Fig. 2).

The validity and reliability of the survey’s questionnaire are used to check its quality (Paul & Maiti, 2008). The reliability is checked with the help of the value of Cronbach’s alpha. The desirable value considered risk-free is that α is greater than or equal to 0.7, and α value around 0.6 is considered acceptable in the exploratory study (Nunnally, 1978). In this study, the α value was found greater than 0.6 for all constructs. This means that questionnaire is reliable.

Two types of validity, convergent and discriminant validity, are used to check the validity of constructs in the survey questionnaire the CFA model calculations. Loadings between measured factors and their items were found greater than 0.5 for all except for PERE1 (0.441). Therefore, the indicators’ loadings for independent variables (Barki & Hartwick, 2001). Most of the loadings in the CFA model were more significant than 0.7, except the few ones: financial support from the associated authorities and regulators (0.590), Compliance with regulatory bodies' regulations and policies (0.543). Industry standards (0.598), productivity after blockchain adoption (0.441), Willingness to accept BLCT adoption risks (0.680), and Active attention and response (0.610). Additionally, three conditions need to be met (Chandra & Kumar, 2021), 1. Standard loading should be more than 0.3; 2. Cronbach’s alpha reliability (CR) value must be at least 0.6; 3. The average variance extracted (AVE) must be at least 0.5. In this study, all these three conditions are met, as shown in Table 2. This refers to the acceptable convergent validity. Tables 2 and 3 show that the square root of each variable's AVE value is more than the correlation of the adjoining variable with the other variables, Which shows acceptable discriminant validity (DV) (Fornell & Larcker, 1981).

**Table 2:** Measurement items, loading factors, Cronbach’s alpha (*α*), Composite reliability (CR), and average variance extracted (AVE)

Construct	Measurement items	Items	Loading	α	CR	AVE
BLCT	Reliability	BLCT1	1.000	0.965	0.944	0.912
	Compatibility	BLCT2	0.979			
	Transparency and traceability	BLCT3	1.325			
	Immutability	BLCT4	1.527			
	Smart contracts	BLCT5	0.961			
	Complexity	BLCT6	0.923			
	Disintermediation	BLCT7	0.930			
	Automation	BLCT8	0.940			
	Verifiability	BLCT9	0.924			
	Auditability	BLCT10	0.926			
	Security and privacy	BLCT11	0.942			
	Acentric database	BLCT12	0.923			
	Provenance	BLCT13	1.067			
GLPR	Efficient resource utilization	GLPR1	1.000	0.962	0.951	0.903
	Reduction in emission	GLPR2	0.957			
	The energy efficiency of process and design	GLPR3	0.797			
	Lean tools and practices	GLPR4	0.973			
	Lean and green technologies	GLPR5	0.943			
SUCI	Understanding the requirement of SC players	SUCI1	1.000	0.964	0.985	0.942
	Information sharing	SUCI2	1.049			
	Collaboration, coordination and strategic alliance	SUCI3	0.830			
	Public, private partnerships	SUCI4	1.210			
	Knowledge and innovation sharing	SUCI5	0.805			
SUCR	Delays in products delivery and pickup	SUCR1	1.000	0.967	0.974	0.911
	Inadequate storage and delivery capacity	SUCR2	0.875			
	Demand volatility	SUCR3	0.927			
	Poor forecasting	SUCR4	0.873			
	Shortage of labor and driver	SUCR5	0.856			
	Road and border closures	SUCR6	0.954			
	Volatile fuel prices	SUCR7	0.836			
IEEC	Availability of required resources	IEEC1	1.000	0.903	0.978	0.813
	Knowledge, experience, and technical expertise	IEEC2	1.043			
	Presence of advanced information sharing	IEEC3	1.028			
	Trust in blockchain	IEEC4	1.044			
	Competitive pressure	IEEC5	0.920			
	Social influence of customs, cultures, and people	IEEC6	0.715			
RESU	Industry standards	RESU1	1.000	0.881	0.961	0.815
	Compliance with regulatory bodies' regulations and policies	RESU2	0.587			
	Adjustment in policies with the market conditions	RESU3	1.304			
	Regulatory environment for data privacy and security	RESU4	0.540			
	Financial support from the associated authorities or regulators	RESU5	0.594			
PERE	Productivity after BC adoption	PERE1	1.000	0.924	0.925	0.911
	Speed of completing tasks/responsiveness	PERE2	1.425			
	Risk reduction	PERE3	0.794			
	Overall quality improvement	PERE4	0.441			
TMSU	Active attention and response	TMSU1	1.000	0.850	0.917	0.814
	Resource (e.g., labor, finances, and materials) accessibility approval	TMSU2	0.854			
	Willingness to accept BC adoption risks	TMSU3	0.669			
	Motivating employees for BC adoption	TMSU4	0.599			
INNC	Application of innovative techniques	INNC1	1.000	0.853	0.925	0.834
	Regular improvement in operations	INNC2	0.976			
	Adoption of innovative and technical solutions	INNC3	0.919			
	Application of standardized and straightforward operations	INNC4	0.820			
	Protection of SC against risks	INNC5	0.931			
CO	Infrastructure cost	CO1	1.000	0.857	0.951	0.816
	Maintenance and operational cost	CO2	0.989			
	Blockchain adoption cost	CO3	0.947			
	Transaction cost	CO4	1.171			
SSCP	SC overall cost	SSCP1	1.000	0.971	0.960	0.937
	Environmental cost	SSCP2	0.985			
	The profitability of sales revenue	SSCP3	0.971			
	Reduction in environmental impact	SSCP4	0.969			
	Reduction in food waste and losses	SSCP5	0.963			
	Empowering farmers and small-scale producers	SSCP6	0.974			
	Number of jobs created	SSCP7	0.946			
	Food safety and security	SSCP8	0.942			
	Stability of the workforce	SSCP9	0.939

**Table 3:** Discriminant validity

	BLCT	IEEC	SUCI	CO	TMSU	PERE	RESU	SUCR	SSCP	INNC	GLPR
BLCT	0.954										
IEEC	0.140*	0.901									
SUCI	0.157**	0.089	0.970								
CO	0.339**	0.291**	0.180**	0.903							
TMSU	0.124*	0.180**	0.220**	0.308**	0.902						
PERE	0.100	0.035	0.028	0.279**	0.092	0.954					
RESU	0.134*	0.124*	0.168**	0.396**	0.245**	0.213**	0.902				
SUCR	0.069	− 0.006	− 0.063	0.083	− 0.058	0.085	0.025	0.954			
SSCP	0.173**	0.000	0.056	0.033	0.105	0.004	0.009	0.175**	0.967		
INNC	0.046	0.003	0.005	0.048	− 0.048	− 0.009	0.021	0.019	0.081	0.913	
GLPR	0.142*	− 0.025	0.119*	0.102	0.245**	0.336**	0.036	− 0.019	0.060	0.050	0.950

*Correlation is significant at the 0.05 level (2-tailed).

**Correlation is significant at the 0.01 level (2-tailed).

### SEM

**Fig. 3:**
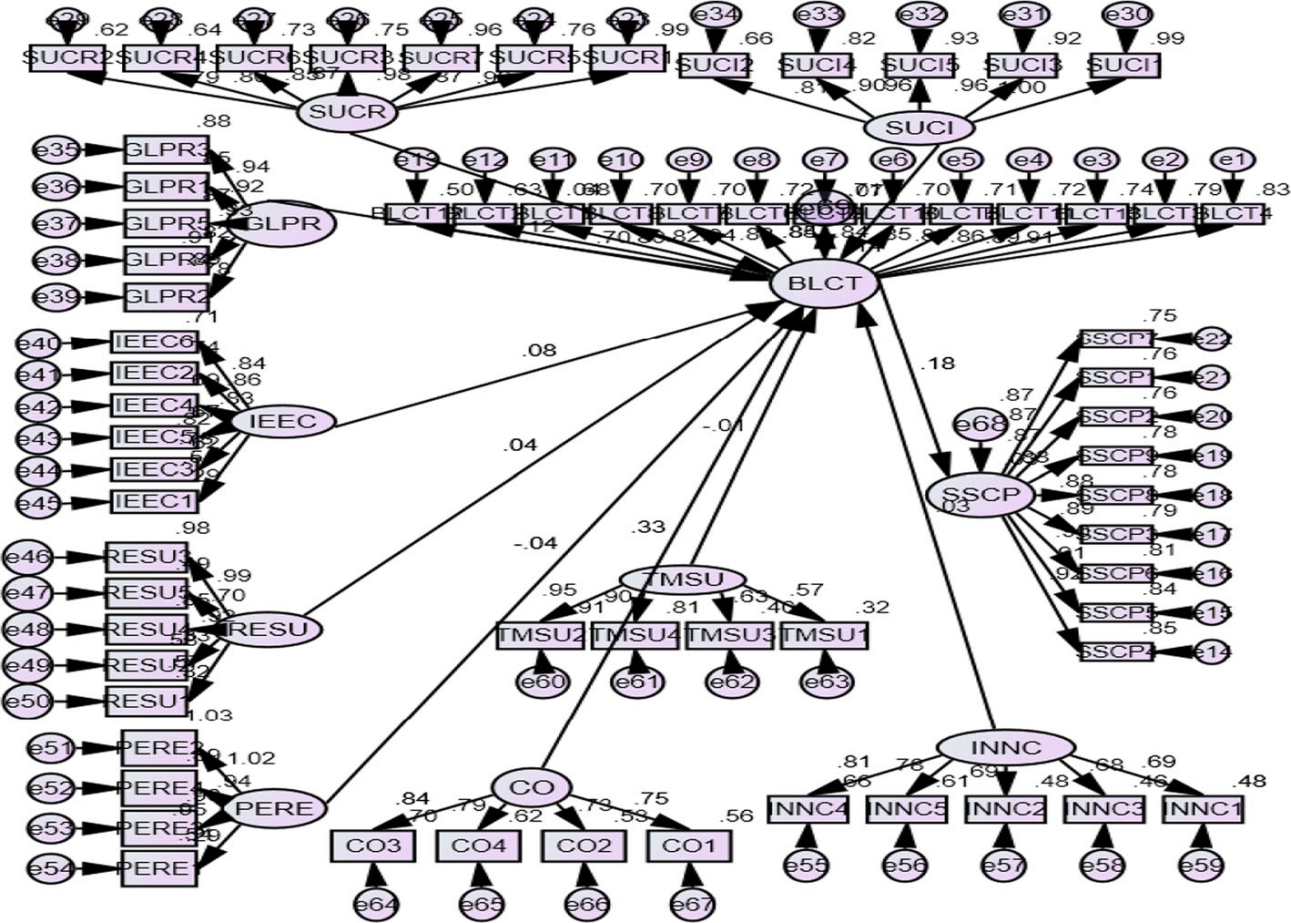
SEM path diagram

A validity test was performed with SEM's help for undiscovered constructs and analysing the fitting model (Chandra & Kumar, 2021). Path study, a specific SEM case, examines the variables' informal and formal relationships to show significant patterns between them. One-directional relations in SEM replace Two-directional relations of the CFA model. Figure 3 (appendix) reveals the Path diagram prepared in AMOS-20.0. Based on the findings obtained, the Chi-square test value was 2.329 (< 3.0), GFI was 0.713, not greater than 0.95.

Additionally, RMSEA (0.066) was more than 0.05, and CFI (0.873) was less than 0.90, but both values were within the acceptable limits. Therefore, Goodness of Fit statistics reveals allowable results for collected data. Table 4 shows the results of path analysis through the SEM model.

**Table 4:** Results of hypotheses testing

Serial number	Hypotheses	Standardized estimates	Supported (Yes/No)	In contrast with	In agreement with
1	GLPR is positively associated with BLCT	0.121	Yes		
2	SUCI is positively associated with BLCT	0.072	Yes	Karamchandani et al. (2020)	
3	SUCR is positively associated with BLCT	0.037	Yes		
4	IEEC is positively associated with BLCT	0.078	Yes		
5	RESU is positively associated with BLCT	0.038	Yes	Wong et al. (2020a)	
6	PERE is positively associated with BLCT	− 0.036	No		Queiroz et al. (2021), Wong et al. (2020b)
7	TMSU is positively associated with BLCT	− 0.012	No		Wong et al. (2020a)
8	INNC is positively associated with BLCT	0.027	Yes		
9	CO is positively associated with BLCT	0.331	Yes		Wong et al. (2020a)
10	BLCT is positively associated with SSCP	0.178	Yes		

Table 4 shows that all nine hypotheses are positively associated with BLCT, and BLCT's relation with sustainable supply chain performance is positively correlated. Path study findings show that eight hypotheses are supported, whereas two are rejected. Our study supports seven hypotheses of the relationship between blockchain technology with Green and Lean Practices (GLPR, 0.121), Supply chain integration (SUCI, 0.072), Supply chain risk (SUCR, 0.037), Internal and External environment conditions (IEEC, 0.078), Regulatory Support (RESU, 0.038), Innovation Capability (INNC, 0.027) and Cost (CO, 0.331). The hypothesis of blockchain technology adoption related to sustainable supply chain performance is also supported (SSCP, 0.178).

The findings of mediation analysis from Table 5 denote that only three mediation effect exists. If form in terms of hypotheses, each of the nine relationships, we can see three hypotheses are accepted in the fully mediating condition where the only indirect effect is working. In some cases, if the only direct effect is working, there cannot be any mediation effect. None of the cases in Table 5 can find direct and indirect effects, leading to partial mediation. Full mediation is occurring for the factors SUCI, GLPR, and CO.

**Table 5:** Results of mediation effect

Relationship	Direct effect	Indirect effect	Result
SUCR→BLCT→SSCP	0.178 (0.002)*	0.009 (0.217)	–
IEEC→BLCT→SSCP	− 0.024 (0.662)	0.008 (0.281)	–
SUCI→BLCT→SSCP	0.031 (0.577)	0.015 (0.057)*	Full mediation
TMSU→BLCT→SSCP	0.119 (0.057)*	− 0.003 (0.609)	–
GLPR→BLCT→SSCP	0.017 (0.707)	0.019 (0.028)*	Full mediation
PERE→BLCT→SSCP	− 0.021 (0.750)	− 0.005 (0.476)	–
INNC→BLCT→SSCP	0.078 (0.179)	0.004 (0.562)	–
RESU→BLCT→SSCP	− 0.021 (0.653)	− 0.001 (0.922)	–
CO→BLCT→SSCP	− 0.064 (0.416)	0.051 (0.006)*	Full mediation

*Significant at α < 0.10 (2-tailed test); *P* values are shown in parentheses.

## Discussion

The mediating role of blockchain technology for sustainable supply chain performance is explored in this study by using SEM statistical methodology. Table 5 reveals the SEM analysis's finding, supporting eight hypotheses, including blockchain technology's relation with the proposed model's sustainable supply chain performance. Eight significant blockchain adoption variables in order of their standardized estimates are Cost (CO), sustainable supply chain performance (SSCP), Green and Lean Practices (GLPR), Environment conditions (IEEC), Supply chain integration (SUCI), Regulatory Support (RESU), Supply chain risk (SUCR), Internal and External and Innovation Capability (INNC).

This study concludes that “Cost” is the essential variable. This is consistent with the results of Wong et al. (2020a). At the same time, Fan et al. (2020) concluded that cost is a conditional variable for blockchain adoption. Unexpectedly, Cost was not supported empirically as a barrier to blockchain technology adoption but a driver for adopting blockchain. The reason can be the advantages that blockchain provides; however, it is perceived as a costly and complicated technology by the users (Wong et al., 2020a; Gallardo et al., 2018). This result does not agree with the previous studies (Shi & Yan, 2016) but agrees with Wong et al. (2020a). The reason for SC cost positively influences BLCT adoption is the reduction of overall cost after implementation. BLCT can remove intermediaries or traders up to some extent in ASC, and distributors can get direct customer payments (Wong et al., 2020a; Chen et al., 2020). The new method of accounting in BLCT shared ledger, less human intervention, real-time monitoring, information accessibility to all players, and smart contract helps in minimizing the paperwork, transaction and food frauds and ASC risks and uncertainties related to climate change, fluctuating supply and demand, etc. (Yadav & Singh, 2020b; Kamilaris et al., 2019). Thus, reducing the cost of transactions, infrastructure, maintenance, operational, and food recall cost.

Green and lean practices construct the second highly significant variable in the model. Keeping in mind the sustainable performance of the supply chain Green and Lean Practices play an essential role in blockchain adoption. Earlier studies have not included green and lean practices as a factor in the blockchain model because they have not explored blockchain's mediating role for sustainability. Raut et al. (2019) included green and lean practices in the SEM-Artificial Neural Network (ANN) model of big-data (BD) analytics for Indian manufacturing firms' sustainability. Green and lean practices support the blockchain technology adoption in our study, which is against the finding of Raut et al. (2019) for BD analytics. In our study, GLPR supports blockchain adoption because Green and lean strategies are supported by adopting at least one Industry 4.0 technology, as Chiarini et al. (2020) discussed through a survey of Italian manufacturing plants. The literature lacks empirical evidence of a relationship between BLCT adoption and GLPR. The improved transparency and real-time monitoring of resource usage through BLCT can help in improved natural resources and suppliers' green performance management. BLCT can also help in the functioning of ASC from farm to fork by using lean and green tools such as value stream mapping (VSM) and life cycle assessment (LCA), thus exposing the spots of food waste and lower green performance.

Internal and external environmental conditions are the third most significant variable in blockchain adoption. Internal and external environmental conditions are facilitating conditions that drive blockchain adoption. Authors in the extant literature have not discussed precisely the effect of internal and external conditions on BLCT adoption. The cause of acceptance of this hypothesis can be explained by discussing items about BLCT adoption. Availability of the right resources, knowledge, technical expertise, and assistance under facilitating conditions positively influences blockchain adoption (Wong et al., 2020b; Queiroz et al., 2021). Competitive pressure also positively influences blockchain adoption because it is necessary for firms to remain competitive (Wong et al., 2020a) and BLCT, a significant technology under industry 4.0. The internal pressure and desire to gain the competitive benefit, upstream and downstream players’ external pressure (Shi & Yan, 2016) in ASC lead to BLCT adoption. The diversity of geographical and economic distribution of SC stakeholders and most ASC players exists in rural areas, thus dominated by customs, culture, and people's hesitation to adopt new technology like BLCT. However, for advanced information technology (IT) that needs to be driven by the industry 4.0 era, BLCT needs to be adopted in ASC to minimize SC risks related to payment fraud, supply management, crop failure, food wastage, etc. The successful execution of BLCT adoption requires knowledge, expertise, and experience of BLCT (Kouhizadeh et al., 2021).

Supply chain integration (SUCI) is the fourth most significant variable concerning blockchain adoption. Supply chain integration hypotheses are in contrast with Karamchandani et al. (2020). SUCI positively influences the relationship between perceived benefits and perceived usefulness of BLCT (Karamchandani et al., 2020). The positive relationship between SUCI and blockchain technology adoption can also be supported by Chiarini et al. (2020) findings for industry 4.0. BLCT provides more transparent, secure, and accurate information sharing than other technology-based traceability systems (Iansiti & Lakhani, 2017). Thus, providing platform to improve collaboration, partnership and strategic alliance performance for efficient knowledge, innovation and information sharing. The BLCT adoption can also help in understanding ASC player's requirements through real-time and accurate information management. To meet the strict food quality and safety requirements, ASC needs to improve SUCI and improve SC visibility. Thus, BLCT can be the technological solution.

Regulatory support is the Fifth most significant variable with blockchain technology adoption. The regulatory support hypothesis agrees with Wong et al. (2020b) but contrasting with Wong et al. (2020a). Wong et al. (2020b) explored the role of regulatory support as a moderator on facilitating conditions' impact on behavioural intention of blockchain adoption. Clearing the regulatory uncertainties overcomes the trust challenge and facilitates the required infrastructure implementation (Wong et al., 2020b). So, the reason behind the acceptance of regulatory hypothesis can be that the managers believe that providing legal certainty to blockchain users through guidance on data protection regulation while using BLCT would develop trust in users for BLCT and data economy by using it for developing transparent SC (Wong et al., 2020b). Shi and Yan (2016) reported that robust policies and laws for BLCT adoption result in its quick adoption. ASC always remains under stringent scrutiny from audit and regulatory bodies on multiple fronts for maintaining the quality and safety of food, and thus firms are pressurized to explore BLCT adoption to improve compliance with regulations. As blockchain can trace the product throughout ASC and provide authentic information on food items (Ghadge & Bourlakis, 2021; Casino et al., 2020).

INNC refers to adapting to the dynamic environment and solving unexpected problems (Wang et al., 2020). Innovation capability positively influences BLCT adoption. A positive relationship can be the need for ASC to mitigate SC risks related to consumer, firm, and environment by improving the supply chain's innovative capability in BLCT enabled supply chain. BLCT can improve innovative capacity to improve SC’s ability to utilize knowledge, products, systems, and ideas for the firm’s advantage based on real-time data availability.

SUCR positively influences BLCT adoption. Choi (2020) found that BLCT enabled SC leads to lower SC risks. The reason for this relationship is BLCT’s capability to mitigate SUCR by efficient inventory and resources management. BLCT mitigates risks related to crop diseases, pest infestations, the uncertainty of supply and demand, and delay of payments due to higher transaction settlement time by reducing human interference, increasing traceability and transparency, improving trust among ASC players, and eliminating intermediaries.

Supply chain risk and Innovation capability are two variables explored by Wang et al. (2020) regarding the relationship between these two in the industry 4.0 era. Innovation capability increase will result in a decrease in customer, company side, and environmental risks. Therefore, firms may adopt new technologies and innovative solutions (Wang et al., 2020). This study's positive relationship between supply chain risks and blockchain adoption is more significant than the positive relationship between innovation capability and blockchain. This can be because those firms are more interested in blockchain adoption for mitigating risks and are more interested in improving innovative capability by other means rather than by only implementing blockchain.

BLCT and SSCP relation is the second most significant hypotheses overall and newly studied. This hypothesis's significant reason can be the capabilities and unique characteristics of BLCT such as traceability, reliability, immutability, smart contracts, automation, disintermediation, security and privacy, decentralized database etc. BLCT capabilities could improve supply chains' sustainable performance by reducing the product recall and rework, food poisoning, actual tracing of carbon footprint, and carbon tax, and efficient resource utilization of soil, water, and energy, thus improving the emission trading system's efficiency and facilitating recycling behaviour (Prashar et al., 2020; Saberi et al., 2018). This helps in reducing food waste and other hazardous waste and emission means achieving environmental sustainability targets. BLCT can help small farmers by supporting insurance programs, protecting labor from exploitation through smart contracts, and ensuring fairness in payments and taxation in ASC (Kamilaris et al., 2019), thus helping achieve social sustainability goals. BLCT is very useful for reducing SC cost in terms of production cost, transportation cost, maintenance cost, food recall cost etc. (Nandi et al., 2020), thus helping in making business operations economically sustainable.

Performance expectancy and top management support are the two variables for which the hypotheses have been rejected in our study. The positive relationship between top management support and BLCT adoption was rejected in our study, which agrees with Wong et al. (2020a). The rejection reason can be that the top management does not have enough knowledge about the advantages of BLCT and thus have a negative intention for BLCT adoption. The relative benefits motivate the top management to support BLCT adoption (Wong et al., 2020a). With the immaturity of BLCT implementation and its regulation as BLCT is a disruptive technology, managers are not convinced about its benefits to ASC in developing countries like India (Kouhizadeh et al., 2021; Wong et al., 2020a, 2020b). The positive relationship of performance expectancy with blockchain adoption was rejected in this study. This agrees with Wong et al. (2020b) and Queiroz et al. (2021). This can be the lack of awareness, knowledge, familiarity, experience, and expertise about blockchain-based systems (Wong et al., 2020b; Queiroz et al., 2021; Kamble et al., 2019). However, BLCT has benefits in reducing transaction cost, production cost, risk management cost, and competitiveness and innovation capability by improving transparency, traceability, information sharing, trust, and collaboration (Stranieri et al., 2021). Stranieri et al. (2021) reported that BLCT does not impact flexibility and responsiveness and does not positively affect product flow management and intrinsic food product quality.

The mediation effect of BLCT for the relationship between SUCI, GLPR, and CO with SSCP proves that BLCT indirectly affects SUCI, GLPR, and CO relation with SSCP. It is evident from this study that GLPR supports BLCT adoption. This study also concludes that BLCT mediates the effect of GLPR on SSCP or indirectly affects the relationship between GLPR and SSCP. The reason for the mediation effect of BLCT in this relationship can be that BLCT promotes GLPR practices which further helps achieve sustainable performance in ASC. BLCT can support green and lean tools such as VSM and LCA by enabling real-time information sharing and monitoring each activity and process in ASC from farm to fork. Green and lean practices help achieve environmental targets and reduce SC's cost by improving resource efficiency, and the saved cost can be utilized for the benefit of society. BLCT helps reduce the cost of SC; thus, cost reduction can support sustainability targets by providing more financial support, which can be utilized to improve resource availability to improve SSCP. Therefore, we can say BLCT indirectly affects the relation of cost with SSCP. BLCT indirectly affects the relation of SUCI with SSCP. BLCT improves SUCI by providing more transparent, secure, and accurate information sharing, thus improving collaboration quality and building trust among players within ASC (Stranieri et al., 2021; Iansiti & Lakhani, 2017). Whereas SUCI helps improve decision-making by supporting the material movement and timely information sharing (Rai et al., 2006). The improved SUCI means improvement in SCP in the forms of quality, delivery cost, and flexibility (Banchuen et al., 2017); thus, SUCI can further enhance SSCP.

### Theoretical implications

This study extends the literature on blockchain technology by empirically exploring the factors affecting blockchain adoption and blockchain technology's role in achieving sustainable performance. A few have explored the role of blockchain technology in sustainable performance. However, in the agri-food sector, there is a lack of good literature on the mediation effect of blockchain technology in between the relationship of blockchain with sustainable supply chain performance empirically. This study prepares the comprehensive base for the feasibility and potential of BLCT adoption for sustainable development in ASC based on UTAUT and TOE theory. This study contributes to the knowledge gap in three ways. First, by exploring the relationship between BLCT and SSCP and finds that BLCT adoption positively affects SSCP. This provides empirical evidence for how BLCT adoption can help in achieving sustainable goals in ASC. Second, this study has also addressed the gap in the relationship of GLPR with BLCT adoption and found that the need for green and lean practices can induce BLCT adoption. This finding highlights that BLCT adoption in ASC can facilitate the successful execution of lean and green tools such as VSM and LCA. Third, the mediating effect of BLCT on the relationship of SUCI, SUCR, IEEC, RESU, PERE, TMSU, INNC, GLPR, and cost with SSCP and found full mediation effect of BLCT for the relation of SUCI, GLPR, and cost with SSCP. This means blockchain indirectly affects SUCI, GLPR, and cost relation with SSCP. In addition to these three new findings, the findings of this study also showed that SUCI, SUCR, IEEC, RESU, INNC, and cost positively affect BLCT adoption.

### Managerial implications

The study provides understanding to the managers, technology service providers, and innovative project handlers about the role of BLCT for improving SP of ASC and the factors that affect BLCT adoption. Cost and green and lean practices are identified as the most significant factors affecting BLCT adoption. Cost is the most critical factor affecting BLCT adoption. Thus, managers need to focus on close coordination with the technology service providers to decrease and compensate for the cost as it requires high computing power and energy or resources, takes a long time for payback, and higher cost of hiring technical specialists because of higher demand (Kamilaris et al., 2019). The implementation cost of BLCT is sustainable as BLCT reduces overall SC cost after successful implementation (Perboli et al., 2018). The cost of BLCT adoption can be compensated by designing a suitable and resilient BLCT application model that can provide maximum benefits to ASC. This study also provides insights that green and lean practices positively influence BLCT and BLCT technology influencing SSCP. Managers can meet their sustainability targets through BLCT by applying green and lean practices and using tools like VSM and LCA. Managers and technology solution providers can utilize BLCT capabilities such as smart contract, immutability, transparency, traceability, trust, disintermediation, automation, verifiability, auditability, shared databased, provenance, and information security for achieving sustainability in SC operations by reducing food waste, food recall cost, food poisoning, and contamination, waste disposal costs, improving consumer health, quality of food delivered and promoting insurance program of small farmers, environmental practices for food production, fair payments thus reducing exploitation of labors. Managers need to improve collaboration, coordination, information-sharing strategy, and strategic alliance among ASC players to accelerate the BLCT adoption. For successful BLCT adoption in ASC, top managers need to focus on existing knowledge, experience, technical expertise, advanced information technology (IT), customs, culture, and people. As ASC mainly exists in rural areas and there is a lack of advanced IT skills dominated by customs and culture, technology service providers need to design BLCT with consideration of extra investment on upgrading existing IT infrastructure and increasing awareness of BLCT benefits to overcome societal pressure. The managers need to consider blockchain as a robust solution for mitigation ASC risk related to uncertainty in supply and demand, crop diseases, climate change, weather conditions, and payment delays as BLCT helps in improving traceability and transparency, increasing trust by eliminating intermediaries and real-time information available and also reducing human interference. This study highlights that the managers need to implement BLCT to improve innovation capability and mitigate SC risks effectively as blockchain helps in utilizing knowledge, products, ideas, and systems efficiently for the firm’s benefit through real-time data availability. The findings reveal that policymakers need to frame robust and specific policies and regulations for the speedy adoption of blockchain. BLCT also helps meet the regulatory requirements of stringent food safety and quality regulations by providing accurate real-time data related to product movement throughout the ASC. After all these benefits, the top management is hesitant and has no trust to support BLCT adoption because of its immaturity and absence of regulations as novel technology. The managers need to be aware of the benefits of BLCT for supporting its adoption in ASC. The technology service providers need to provide cost-effective and innovative BLCT design and increase awareness by providing successful implementation examples and their relative benefits in profitability and durability.

Additionally, managers need to be aware of the indirect effect of BLCT for the relationship of SUCI, GLPR, and cost with SSCP. This means BLCT somehow supports SUCI, GLPR, and cost to enhance SSCP further. Therefore, this study provides implications for both managers and service providers.

## Conclusion and future scope

Our study discovered the mediating role of blockchain technology to improve the SSCP of the agri-food sector in Indian ASC. However, authors have previously discussed blockchain's critical success factors for sustainability and blockchain adoption factors separately. This study explained blockchain's mediating role in achieving agriculture supply chain sustainability based on UTAUT and TOE theory on BLCT adoption. The proposed research model on BLCT identifies nine factors: GLPR, SUCI, SUCR, IEEC, RESU, PERE, TMSU, INNC, and cost that influence BLCT adoption. The proposed model was validated by performing a survey in Indian ASC, and an overall sample from 316 respondents was collected. The findings suggest that SUCI, GLPR, and cost need mediating support of BLCT to affect SSCP.

For mitigating supply chain risks caused by climate change, crop diseases, supply and demand uncertainties, and the long payback period, BLCT adoption can be useful. The innovation capability of ASC can also be improved through blockchain adoption. Cost, green and lean practices, and internal and external environmental conditions are the most significant to affect blockchain adoption, respectively. The supply chain cost incurred for blockchain implementation is higher due to high resource requirements (Wong et al., 2020a), but the benefits that BLCT provides can reduce the overall cost of SC after its use in ASC. This study suggests that top managers need to be aware of the benefits that BLCT provides for efficiently managing supply chain operations and improving performance. The reason for low awareness and knowledge of BLCT benefits are its immaturity of implementation, lack of empirical evidence of performance benefits, and absence of robust and specific legislations of its use and requirement of regulations update. Overall, cost and green and lean practices play an essential role in affecting blockchain adoption, and these factors further improve SSCP with the help of the indirect effect of BLCT. This study assists stakeholders, managers, policymakers, technology service providers, and innovative project handlers in understanding and identifying the role of critical factors affecting BLCT adoption and additionally, forming strategies for improving sustainable performance by improving SC integration, green and lean practices implementation, and reducing cost of SC and also through blockchain adoption.

### Limitations and future scope

Researchers can target future work as follows: (1) The role of other factors based on TAM theory can be explored with the same models. (2) GLPR, IEEC, and SUCR effect on SSCP can be explored without mediation effect of BLCT as these relations were identified as gaps in literature (3) The supply chain risk factor can be categorized as different factors, namely consumer side risk, environmental risks, and firm side risks (Wang et al., 2020) for further extension of the model. (4) Top management support and SC integration can be explored as moderators in the same model. (5) Trust in technology can also be included as the model's left side variable by making necessary changes in the items. (6) Internal and external environmental conditions can be taken as separate variables.

## Appendix

See Table 6.

**Table 6:** Proposed factors and sub-factors

Sr.No.	Factors	Sub-factors/item	Author & year	Description
1	Green and lean practices (GLPR)	Efficient resource utilization (GLPR1)	Chiarini et al. (2020), Raut et al. (2019), Muñoz-Villamizar et al. (2019)	The combined concept of lean and green helps reduce waste, air pollution, and solid hazardous waste from the agri-food supply chain operations from farm to fork. Value steam mapping (VSM) and life cycle assessment tool (LCA) for green and lean management is mainly used to eliminate waste by exposing it and increasing the sustainable efficiency of agri-processes.
		Reduction in emission (GLPR2)		
		The energy efficiency of process and design (GLPR3)		
		Lean tools and practices (GLPR4)		
		Lean and green technologies (GLPR5)		
2	Supply chain integration (SUCI)	Understanding the requirement of SC players (SUCI1)	Chiarini et al. (2020), Raut et al. (2019), Singh et al. (2019), Mangla et al. (2018)	Information sharing, resource sharing, collaboration, coordination, knowledge sharing of efficient strategies can improve the efficiency of each ASC activity such as farm operations, procurement, processing, distribution, and sales. For a sustainable ASC, firms need to involve suppliers and consumers to meet stringent food quality and safety regulations.
		Information sharing (SUCI2)		
		Collaboration, coordination, and strategic alliance (SUCI3)		
		Public, private partnerships (SUCI4)		
		Knowledge and innovation sharing (SUCI5)		
3	Supply chain risk (SUCR)	Delays in products delivery and pickup (SUCR1)	Wang et al. (2020)	The ASC is vulnerable to risks such as crop diseases and pest infestations due to uncertain weather conditions that need to be mitigated and controlled. The ASC also faces issues related to higher transaction settlement period as it includes labor-intensive activities and involvement of small holding farmers
		Inadequate storage and delivery capacity (SUCR2)		
		Demand volatility (SUCR3)		
		Poor forecasting (SUCR4)		
		Shortage of labor and driver (SUCR5)		
		Road and border closures (SUCR6)		
		Volatile fuel prices (SUCR7)		
4	Internal and external environment conditions (IEEC)	Availability of required resources (IEEC1)	Kouhizadeh et al. (2021), Wong et al. (2020a, 2020b), Narwane et al. (2020), Queiroz et al. (2021), Karamchandani et al. (2020), Kim and Shin (2019)	Agricultural firms need to define BLCT adoption policies related to proper usage. ASCs includes many customs because of its maximum existence in rural area and producers still use conventional farming. This can cause opposition and hesitation from SC players also. The diversity of geographical and economic distribution of SC stakeholders affects privacy needs and the economic value of data at which they will be ready to share information. The current ASC is conventional and needs to be updated regarding machinery and facilities related to the latest advanced technology and process supporting BLCT adoption and sustainable supply chain (SSC) by gathering real-time information. The resources available to knowledge and expertise, such as internet connectivity at rural and urban agricultural sites and skilled labour, may affect both SSC and BLCT.
		Knowledge, experience, and technical expertise (IEEC2)		
		Presence of advanced information sharing (IEEC3)		
		Intention to adopt blockchain (IEEC4)		
		Competitive pressure (IEEC5)		
		Social influence of customs, cultures, and people (IEEC6)		
5	Regulatory support (RESU)	Industry standards (RESU1)	Wong et al. (2020a, 2020b), Govindan et al. (2016)	RESU is considered as a medium to eliminate the problem of trust among SC players in ASC. The RESU provides legal certainty to the users of BLCT by implementing guidelines related to data protection and its use for transparent SC processes. This will also improve the trust of ASC players in BLCT use. The specific and robust policies and laws for BLCT adoption in compliance with regulatory bodies and industry standards and adjusted to market conditions can quickly adapt. For quick adoption of BLCT, concerned regulatory bodies should financially support the policy makers of BLCT.
		Compliance with regulatory bodies' regulations and policies (RESU2)		
		Adjustment in policies with the market conditions (RESU3)		
		Regulatory environment for data privacy and security (RESU4)		
		Financial support from the associated authorities or regulators (RESU5)		
6	Performance expectancy (PERE)	Productivity after BLCT adoption (PERE1)	Stranieri et al. (2021), Queiroz et al. (2021), Helo and Hao (2019)	This refers to the performance measures which are believed to be achieved after adopting blockchain and thus affect the intention to adopt blockchain. BLCT can improve the traceability of the system, which may also enhance perception about food product information, so the consumers can be ready to pay more for the products. Increased transparency due to improved information sharing for better management of vertical relationships among ASC players reduces transaction costs, thus can improve SCP. Improved traceability can also increase profits by maintaining stable production costs, food quality, and safety. However, flexibility and responsiveness within the ASC is not impacted by the BLCT adoption. The improved collaboration among SC stakeholders due to overcoming trust issues through BLCT helps develop competencies in competitive advantage, innovation, and the ability to identify weaknesses and handle it.
		Speed of completing tasks/responsiveness (PERE2)		
		Risk reduction (PERE3)		
		Overall quality improvement (PERE4)		
7	Top management support (TMSU)	Active attention and response (TMSU1)	Wong et al. (2020a, 2020b)	This factor refers to top management's role in making decisions related to initiation, providing resources for blockchain adoption, and encouraging and motivating the employees. The insufficient knowledge of BLCT and its benefits and legislations are responsible for the low interest of top managers in inventing it and developing required skills. In ASC, there is a lack of empirical evidence on the BLCT adoption benefits in food safety and quality, inventory control, responsiveness, and risk management related to uncertainty like climate change and weather conditions, natural calamities, pest attacks etc.
		Resource (e.g., labor, finances, and materials) accessibility approval (TMSU2)		
		Willingness to accept BLCT adoption risks (TMSU3)		
		Motivating employees for BLCT adoption (TMSU4)		
8	Innovation capability (INNC)	Application of innovative techniques (INNC1)	Wang et al. (2020)	It means a firm's ability to apply simple, creative, standardized, innovative solutions and technologies to improve ASC and protect it against risks related to climate, natural calamities, diseases etc. The innovative technique should provide real-time monitoring of all ASC players and bring them to one platform through a shared database. BLCT helps improve operations at regular intervals through continuous tracking of the event throughout the ASC from farm to fork. Thus, improving food safety, quality, reducing risks, and lead time leads to improved service and higher consumer satisfaction by delivering good quality products at a time.
		Regular improvement in operations (INNC2)		
		Adoption of innovative and technical solutions (INNC3)		
		Application of standardized and straightforward operations (INNC4)		
		Protection of SC against risks (INNC5)		
9	Cost (CO)	Infrastructure cost (CO1)	Wong et al. (2020a), Yadav and Singh (2020b), Ivanov et al. (2019), Hofmann et al. (2018)	This factor refers to the overall cost of SC from hardware, software, labor, maintenance, distribution, transportation, transaction to BLCT adoption cost. The reduction of human error due to its implementation also reduces costs at retailer and manufacturer’s ends. It also reduces administrative, infrastructure or facility, manpower, operational, energy, and maintenance costs due to lowered investment in paperwork, consumable items, time-saving, speedy decision making, improved management and administration, and shared database. BLCT enabled ASC to mitigate risks by efficiently managing demand and supply, thus reducing inventory management costs. Cryptographic signature protection and smart contracts may help prevent suppliers and other ASC players from frauds and ensure safe and secure transactions, thus reducing transaction costs. The improved traceability of BLCT enabled ASC leads to reduced SC cost by increasing responsiveness and assuring quality and safety of delivered food products. The cost incurred by SC risk such as climate change, riots, pandemic, order delay and missing, machine breakdown can also be reduced through BLCT adoption.
		Maintenance and operational cost (CO2)		
		Blockchain adoption cost (CO3)		
		Transaction cost (CO4)		
10	Blockchain technology (BLCT)	Trust Or Reliability (BLCT 1)	Stranieri et al. (2021), Queiroz et al. (2021), Wong et al. (2020a), Chang et al. (2020), Kamble et al. (2020), Prashar et al. (2020), Kumar et al. (2020), Queiroz and Wamba (2019), Wang et al. (2019b), Salah et al. (2019), Zhao et al. (2019), Kim and Shin (2019)	Smart contracts, real-time management of all SC activities and transactions helps in improving trust among SC partners and participation of smallholders. The immutability benefits of BLCT adoption is responsible for trust enhancement and a transparent system for improved traceability in ASC. BLCT eliminates intermediaries and ensures SC traceability and transparency in ASC to reduce risks by providing a decentratlized platform to all players of ASC to access authentic real-time information of product at any time and also track food items. The increased automation and common platform for food prices' bargaining help distributors eliminate intermediaries or traders through direct payment to customers. The provenance capability of BLCT provides ASC the last mile connectivity, and all the players can trace back the orgin of the food item througot the chain. BLCT accompanied by shared database, digital tokens and unique fingerprints for each food product ensures suitability and verifiability of information. The uncertainty of return on investment and dominance of traditional solutions can affect the capability of BLCT in ASC. The real-time monitoring of food products’s movements and custody promotes transparency in ASC. The common ledger for information storage and access can put the privacy of ASC firms at risk. The validation requirement for each transaction in BLCT enabled ASC to assure no change in information after storage, saving from fraud and ensuring information security.
		Compatibility (BLCT 2)		
		Transparency and traceability (BLCT 3)		
		Immutability (BLCT 4)		
		Smart contracts (BLCT 5)		
		Complexity (BLCT 6)		
		Disintermediation (BLCT 7)		
		Automation (BLCT 8)		
		Verifiability (BLCT 9)		
		Auditability (BLCT 10)		
		Security and privacy (BLCT 11)		
		Acentric database (BLCT 2)		
		Provenance (BLCT 13)		
11	Sustainable supply chain performance (SSCP)	SC overall cost (SSCP1)	Dubey et al. (2020), Prashar et al. (2020), Kamilaris et al. (2019), Zhao et al., (2019), Wang et al. (2019a), Helo and Hao (2019), El Bilali and Allahyari (2018), Allaoui et al. (2018), Tsang et al. (2018)	In BLCT enabled SC, the availability of comprehensive data related to the shelf life of food can be used for food waste reduction. Firms can also reduce food poisoning, food recall, food spoilage, and food contamination by real-time monitoring of food through BLCT, thus reducing waste disposal and degradation to the environment by improving natural resource management. The improved traceability also helps in improving consumer health and the quality of products delivered to them in compliance with regulations. BLCT can also promote insurance programs for securing farmers against risks such as natural disasters and climate change. It can also help in raising awareness about environmental practices followed for food production. BLCT based contracts can help in reducing the exploitation of labor by assisting authorities in fairness in payments and taxation. BLCT can also tackle corruption and the insufficient environmental, social, and economic regulations in developing countries.
		Environmental cost (SSCP2)		
		The profitability of sales revenue (SSCP3)		
		Reduction in environmental impact (SSCP4)		
		Reduction in food waste and losses (SSCP5)		
		Empowering farmers and small scale producers (SSCP6)		
		Number of jobs created (SSCP7)		
		Food safety and security (SSCP8)		
		Stability of the workforce (SSCP9)		

See Figs. 2 and 3.
